# Association between maternally perceived quality and pattern of fetal movements and late stillbirth

**DOI:** 10.1038/s41598-019-46323-4

**Published:** 2019-07-08

**Authors:** Billie F. Bradford, Robin S. Cronin, Lesley M. E. McCowan, Christopher J. D. McKinlay, Edwin A. Mitchell, John M. D. Thompson

**Affiliations:** 1 Department of Obstetrics and Gynaecology, Faculty of Medical and Health Sciences, University of Auckland, Auckland, New Zealand; 2 Department of Paediatrics: Child and Youth Health, Faculty of Medical and Health Sciences, University of Auckland, Auckland, New Zealand; 3 Liggins Institute, University of Auckland, Auckland, New Zealand; 4 Kidz First Neonatal Care, Counties Manukau Health, Auckland, New Zealand

**Keywords:** Epidemiology, Patient education

## Abstract

We investigated fetal movement quality and pattern and association with late stillbirth in this multicentre case-control study. Cases (n = 164) had experienced a non-anomalous singleton late stillbirth. Controls (n = 569) were at a similar gestation with non-anomalous singleton ongoing pregnancy. Data on perceived fetal movements were collected via interviewer-administered questionnaire. We compared categorical fetal movement variables between cases and controls using multivariable logistic regression, adjusting for possible confounders. In multivariable analysis, maternal perception of the following fetal movement variables was associated with decreased risk of late stillbirth; multiple instances of ‘more vigorous than usual’ fetal movement (aOR 0.52, 95% CI 0.32–0.82), daily perception of fetal hiccups (aOR 0.28, 95%CI 0.15–0.52), and perception of increased length of fetal movement clusters or ‘busy times’ (aOR 0.23, 95%CI 0.11–0.47). Conversely, the following maternally perceived fetal movement variables were associated with increased risk of late stillbirth; decreased frequency of fetal movements (aOR 2.29, 95%CI 1.31–4.0), and perception of ‘quiet or light’ fetal movement in the evening (aOR 3.82, 95%CI 1.57–9.31). In conclusion, women with stillbirth were more likely than controls to have experienced alterations in fetal movement, including decreased strength, frequency and in particular a fetus that was ‘quiet’ in the evening.

## Introduction

Maternal perception of decreased fetal movements (DFM) is a frequently encountered problem in maternity care, with between 4 and 16% of women presenting with DFM^1,2^. Perception of altered or decreased fetal movements is associated with stillbirth^3^ and with other adverse outcomes including; feto-maternal haemorrhage, cord accident, oligohydramnios, congenital anomaly, being small for gestational age (SGA) and long term neurodevelopmental impairment^3–5^. DFM is hypothesised to be a compensatory response to placental insufficiency, allowing the fetus to conserve energy, and therefore may provide early warning of deteriorating fetal condition^6^.

Several studies have concluded that suboptimal care in relation to DFM contributes to potentially avoidable stillbirth^7,8^. However, the optimal management of women presenting with DFM has not been established. Women with fetal movement concerns need to present early enough for intervention to be possible, if appropriate. However, use of interventions must be balanced against the potential for harm if such interventions are too liberally applied. In Norway, introduction of a fetal movement awareness quality-improvement programme resulted in a significant reduction in stillbirths amongst women with DFM, without increasing overall presentations for DFM or inductions of labour^9^. The authors attributed the decrease in stillbirths to a reduction in delayed presentation with DFM (>24 hours and >48 hours) and increased identification of fetal growth restriction via ultrasound scanning of all women with DFM. Subsequently, a large trial in the United Kingdom (AFFIRM) investigated a package of care that included encouraging awareness of fetal movements and a management plan involving a low threshold for inducing labour in women with DFM. In the AFFIRM trial there was an increase in induction of labour and prolonged neonatal unit stays with no significant reduction in stillbirths^10^. However, data on the frequency of DFM consultations, delayed presentation with DFM and utilisation of ultrasound scans were not reported. Compliance with the management protocol was sub-optimal with up to 40% of hospitals involved citing difficulties complying with the scanning recommendations.

Giving women information about fetal movements can increase maternal concern^11^, and as the AFFIRM trial has demonstrated potentially lead to unnecessary interventions^10^. An optimal approach to fetal movement screening would ensure timely presentation in women with a fetus at risk but avoid creating excessive concern in women with a healthy fetus. Clinical practice guidelines emphasise the importance of acknowledging women’s subjective concerns about a change in fetal movements rather than any fetal movement count per se^3^. Qualitative aspects of fetal movements such as, pattern and intensity are observed by pregnant women but very little is known about these features in relation to stillbirth. Improved understanding of alterations in pattern and intensity of perceived fetal movements in the compromised fetus could enable development of tailored interventions aimed to prevent stillbirth and avoid unnecessary intervention.

Thus, we undertook a case-control study to explore the relationship between maternal perception of fetal movement quality and pattern and late stillbirth. We hypothesised that late stillbirth would be associated not only with perceived changes in fetal movement strength and frequency, but also differences in maternal perception of (1) fetal movement clusters or ‘busy times’ and (2) fetal movement quality in relation to diurnal rhythm, maternal position and meals.

## Methods

Ethical approval was obtained from the Northern X Regional Ethics Committee: NTX/06/05/054, and all methods were performed in accordance with the processes outlined in the approved study protocol. All participants provided written informed consent. A detailed protocol is available online on figshare doi: 10.17608/k6.auckland.3483134.v1.

**Figure 1:**
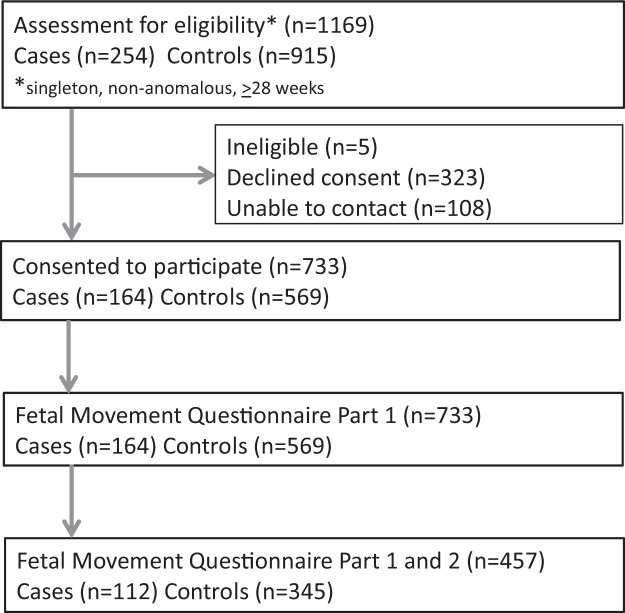
Participant Flowchart.

The study was conducted across seven of twenty health regions in New Zealand, which together account for two-thirds of New Zealand’s approximately 60,000 births each year. We planned to recruit two controls for each estimated eligible case in each region. Recruitment occurred between February 2012 and December 2015 (see Fig. 1). Eligible participants were approached by their midwife or doctor and asked whether the research midwife could contact them to explain the study and invite them to participate. Women with multiple pregnancy or babies with major congenital anomaly were excluded.

Cases were women who had experienced a singleton late stillbirth (≥28 weeks’ gestation) and were interviewed as soon as possible after their stillbirth and before six weeks postpartum. Controls were women with ongoing singleton non-anomalous pregnancies randomly selected from hospital booking lists based on frequency matching of the expected distribution of stillbirths by region and gestation, ensuring that controls would be at a similar gestation to stillbirth cases. In addition to questions about fetal movements, the structured interview included a range of questions about lifestyle factors such as diet and sleep. Eligible participants were informed that the broad aim of the study was to explore modifiable risk factors for stillbirth. The specific hypotheses under investigation were not discussed with participants. The methods for this study have been described previously^12^.

Participants responded to questions on fetal movement strength, frequency, hiccups, unusually vigorous movements and uterine contractions pertaining to the two weeks prior to stillbirth, or prior to interview for controls. Data were collected face-to-face via a structured interview questionnaire administered by trained research midwives. Interviews were conducted in the location of the woman’s choosing, in most cases the woman’s home and lasted approximately one hour. The structured questionnaire was supplemented in July 2013 (Part 2) with further questions on perception of fetal movement clusters or ‘busy times’, and fetal movement strength in relation to: maternal position and activity, time of day, consumption of food and drinks, and environmental stimuli, such as loud noises and touch.

Maternal perception of fetal movement strength and frequency over the last two weeks was categorised as ‘increased’, ‘decreased’, ‘stayed the same’ or ‘unsure’. If participants were unsure, the fetal hiccups sensation was described as ‘regular jerking movements happening at 1–2 second intervals over a period of 1–2 minutes’. Busy times were defined for participants as ‘a period where there is a group of movements, rather than single isolated movements, which might be short (15–45 seconds), or prolonged and involving many movements for up to 20 minutes.’ Participants indicated how many times a day, on average, their baby had busy times. Changes in duration of busy times in the last two weeks were categorised as ‘longer than before’, ‘about as long as before’ or ‘shorter than before’. Fetal movement quality in relation to maternal position, activities, and time of day was categorised as ‘notably quiet’, ‘subtle or light movement’, ‘moderate movement’, ‘strong movement’, ‘jumps or startles’ and ‘unsure/don’t notice’.

Decreased frequency and decreased strength were significantly associated with each other and could not be used together in a multivariable model. In order to further explore relationships between strength, frequency and stillbirth we developed a set of 16 combined strength and frequency variables, prioritised according to distribution of the most common response in the control group and similarity of risk between variables, in the same manner reported by Heazell *et al*.^13^. Including each of the 16 potential combinations of strength and frequency changes as an independent variable in a multivariable model would have greatly reduced statistical power. These strength/frequency prioritised variables were then combined in a multivariable model with frequency of hiccups and unusually vigorous movements, as well as potential confounders; gestation, parity, employment, maternal body mass index (BMI) and infant sex.

Categorical fetal movement variables were compared between cases and controls, odds ratios were estimated using logistic regression to determine the effect size of any association with late stillbirth. In multivariable analysis, we included any variables that were significant at the 10% level in the univariable analysis and with less than 10% missing data. Estimates of late stillbirth risk are presented as adjusted odds ratios (aOR) with 95% confidence intervals (95% CI), with significance defined at the 5% level. Statistical analysis was carried out using SAS version 9.4 (SAS institute Inc., Cary, NC, USA).

## Results

In total, 733 women were enrolled in this study: 164 women with a late stillbirth and 569 control women with ongoing pregnancies matched for gestation and locality. This recruitment rate represents 65.9% of cases and 62.2% of eligible controls. Causes of stillbirth and sociodemographic associations in this study have been reported elsewhere^12^. Briefly, stillbirth was associated with higher maternal BMI, maternal age >40 years, and SGA (birthweight <10^th^ centile customised). The median (IQR) gestation at time of stillbirth was 37.7 (34.1, 39.9) weeks compared to time of interview for controls of 37.4 (34.0, 38.9) weeks (p = 0.003). The median (interquartile range, [IQR]) duration from estimated time of stillbirth to interview was 24 (16, 32) days. A subgroup of women (those enrolled between July 2013 and December 2015) who completed both Part 1 and 2 of the fetal movement questionnaire that included the additional questions described above (112/164 [70%] cases; 345/569 [61%] controls). Characteristics of this subgroup were broadly similar to the study population as a whole (Table 1).

**Table 1:** Participant characteristics.

Characteristic	Cases N = 164			Controls N = 569		
	Part 1 and 2 of questionnaire N = 112	Part 1 of questionnaire only N = 52	p	Part 1 and 2 of questionnaire N = 345	Part 1 of questionnaire only N = 224	p
**Age (years)**						
<20	4 (3.6)	5 (9.6)	0.28	5 (1.4)	12 (5.4)	0.03
20–39	98 (87.5)	43 (82.7)		328 (95.1)	204 (91.1)	
≥40	10 (8.9)	4 (7.7)		12 (3.5)	8 (5.6)	
**Ethnicity**						
Māori	21 (18.7)	5 (9.6)	0.33	33 (9.6)	25 (11.2)	0.02
Pacific	23 (20.5)	15 (28.8)		46 (13.3)	40 (17.9)	
Indian	12 (10.7)	5 (9.6)		57 (16.5)	20 (8.9)	
Other Asian	11 (9.8)	2 (3.8)		38 (11.0)	34 (15.2)	
European	41 (36.6)	24 (46.1)		166 (48.1)	97 (43.3)	
Other	4 (3.6)	1 (1.9)		5 (1.4)	8 (1.4)	
**Parity**						
0	53 (47.32)	23 (44.2)	0.82	147 (42.6)	98 (43.7)	0.33
1–3	53 (47.3)	27 (51.9)		191 (55.4)	117 (52.2)	
≥4	6 (5.4)	2 (3.8)		7 (2.0)	9 (4.0)	
**BMI (booking)**						
<25	43 (38.4)	19 (36.5)	0.29	177 (51.3)	113 (50.4)	0.84
25–29.9	32 (28.6)	10 (19.2)		89 (25.8)	55 (24.5)	
≥30	37 (33.0)	23 (44.2)		79 (22.9)	56 (41.5)	
**Smoking**						
Yes	22 (19.6)	15 (28.8)	0.19	31 (9.0)	35 (15.6)	0.01
**Employment (last month)**						
Paid work	51 (45.95)	22 (42.3)	0.66	211 (61.7)	121 (55.2)	0.13
**Gestation**						
Preterm	52 (46.4)	16 (30.8)	0.06	160 (46.4)	92 (41.1)	0.21
Term	60 (53.6)	36 (69.2)		185 (53.6)	132 (58.9)	
**Socioeconomic deprivation quintile**						
1–2	23 (20.5)	17 (32.7)	0.23	127 (36.8)	80 (35.7)	0.95
3	25 (22.3)	9 (17.3)		65 (18.8)	44 (19.6)	
4–5	64 (57.1)	26 (50.0)		153 (44.3)	100 (44.6)	

Data are n (%). FM, fetal movement; BMI, body mass index (kg/m^2^). Quintiles 4 and 5 are the most socioeconomically deprived. Part 1 of the questionnaire was completed by all participants interviewed between February 2012 and December 2015. Part 1 and Part 2 was completed by those interviewed between July 2013 and December 2015.

In women with stillbirth, the most common reason for seeing a health professional at the time of diagnosis of fetal death was DFM (n = 64, 39%), followed by labour (n = 44, 27%). In 17% of cases, the fetal death was discovered at a routine antenatal visit (n = 28). We compared maternally perceived fetal movements in the two weeks prior to the stillbirth or to the interview for gestation-matched controls. The majority of controls (58.1%) reported increased strength of fetal movements in the last two weeks. In univariable analysis, maternal perception of increased strength or increased frequency of movements in the last two weeks was associated with reduced odds of late stillbirth, whilst decreases in perception of strength or frequency were associated with stillbirth (Table 2). Maternal perception of fetal hiccups was found to be associated with reduced odds of stillbirth, but uterine contractions showed no association. Analysis of paired strength and frequency variables showed that compared to ‘stay the same’ odds of late stillbirth were increased when both strength and frequency of fetal movements were decreased (OR 2.88, 95% CI 1.62–5.11) (Supplementary Table 1).

**Table 2:** Univariable analysis of fetal movement strength, frequency and odds of late stillbirth.

	Cases N = 164	Controls N = 569	Odds Ratio (95% Confidence Interval)	p
**In the last two weeks did the strength of your baby’s movements?**				
Increase	32 (19.5)	330 (58.1)	0.22 (0.14–0.34)	<0.0001
Decrease	47 (28.6)	45 (7.9)	2.35 (1.44–3.82)	
Stay the same	80 (48.8)	180 (31.7)	reference	
Unsure	5 (3.0)	13 (2.3)	0.86 (0.29–2.51)	
**In the last two weeks did the frequency of your baby’s movements?**				
Increase	21 (12.8)	221 (38.8)	0.31 (0.18–0.52)	<0.0001
Decrease	62 (37.8)	83 (14.6)	2.41 (1.59–3.36)	
Stay the same	79 (48.2)	255 (44.8)	reference	
Unsure	2 (1.2)	10 (1.7)	0.65 (0.14–3.01)	
**During the last two weeks did you notice any time that your baby was more vigorous than usual?**				
Yes	67 (40.9)	330 (58.0)	0.50 (0.35–0.71)	<0.0001
No	97 (59.1)	239 (42.0)	reference	
**During the last two weeks did you feel you baby having hiccups?**				
Yes	77 (47.5)	406 (71.5)	0.39 (0.26–0.57)	<0.0001
No	68 (41.9)	140 (24.6)	reference	
Unsure	17 (10.5)	22 (3.8)	1.59 (0.78–3.19)	
**During the last two weeks did you feel uterine contractions (tightenings/pre-labour contractions/Braxton Hicks contractions/false labour) for longer than an hour?**				
Yes	67 (41.1)	227 (39.9)	1.05 (0.74–1.50)	0.93
No	93 (57.1)	332 (58.4)	reference	
Unsure	3 (1.8)	9 (1.6)	1.19 (0.31–4.48)	

Where column numbers do not add up to N this is due to missing data.

In multivariable analysis, increased strength of movements was associated with reduced odds of late stillbirth (aOR 0.21, 95% CI 0.12–0.36) (Table 2). Decreased frequency was associated with increased odds of late stillbirth (aOR 2.14, 95% CI 1.25–3.67) (Table 3). Multiple episodes of more vigorous than usual movements were associated with reduced odds of late stillbirth (aOR 0.52, 95% CI 0.32–0.84). Daily (aOR 0.28 95% CI 0.15–0.52) or occasional (aOR 0.48 95% CI 0.29–0.80) perception of fetal hiccups (Table 3) was associated with reduced odds of late stillbirth.

**Table 3:** Multivariable analysis of strength and frequency of fetal movements, and odds of late stillbirth.

Fetal movement variable	Cases N = 164	Controls N = 569	Univariable Odds Ratio (95% CI)	p	*Multivariable Odds Ratio (95% Confidence Interval)	p
**Combination of strength frequency changes (prioritised variable)**						
Increased strength	32 (19.5)	330 (58.0)	0.21 (0.13 = 0.33)	<0.0001	0.21 (0.12–0.36)	<0.0001
Increased frequency but not strength	6 (3.6)	29 (5.1)	0.67 (0.30–1.50)		0.65 (0.25–1.71)	
Decreased frequency	56 (34.1)	58 (10.2)	2.08 (1.30–1.5)		2.14 (1.25–3.67)	
Unsure strength or frequency	3 (1.8)	13 (2.3)	0.46 (0.13–1.67)		0.40 (0.10–1.62)	
Same strength or frequency	64 (39.0)	138 (24.2)	reference		reference	
**During the last two weeks did you notice any time that your baby was more vigorous than usual?**						
No	97 (60.2)	239 (42.6)	reference	<0.0001	reference	0.006
Once	22 (13.6)	32 (6.1)	1.59 (0.89–2.36)		1.72 (0.85–3.46)	
More than once	40 (24.8)	280 (49.9)	0.35 (0.23–0.53)		0.52 (0.32–0.84)	
Unsure	2 (1.2)	8 (1.4)	0.62 (0.13–2.95)		0.91 (0.13–6.33)	
**How often did you feel your baby having hiccups in the last two weeks?**						
None	68 (41.9)	140 (24.6)	reference	<0.0001	reference	<0.0001
Unsure if felt	17 (10.6)	22 (3.9)	1.59 (0.79–3.19)		1.95 (0.81–4.67)	
Once	10 (6.2)	22 (3.9)	0.94 (0.42–2.09)		1.04 (0.40–2.70)	
Occasionally	42 (26.2)	197 (35.3)	0.44 (0.28–0.80)		0.48 (0.29–0.80)	
Daily	20 (12.5)	165 (29.6)	0.25 (0.14–0.43)		0.28 (0.15–0.52)	
Unsure	3 (1.9)	11 (1.9)	0.56 (0.15–2.08)		0.72 (0.17–3.50)	

Data are n (%). *Adjusted for parity, employment, body mass index, fetal sex, and gestation. All fetal movement variables in the table are included in the model. Where column numbers do not add up to N this is due to missing data.

In data related to fetal movement quality and pattern included in Part 2 of the questionnaire, ‘notably quiet’ had few responses and was associated with similar odds of late stillbirth as ‘subtle or light movement’. Likewise, ‘jumps or startles’ had very few responses and was associated with similar odds as ‘strong movement’. Therefore, these categories were combined to create a ‘quiet or light movement’ category and a ‘strong or jumps/startles’ category. Multivariable models for variables associated with fetal movement quality and pattern were developed separately to those of types of movement, due to the reduced sample size and the strong inter-relationships of these variables.

The number of busy times perceived per day did not differ significantly between cases and controls (p = 0.13) in univariable analysis (Supplementary Table 2). However, busy times that were ‘shorter than before’ were associated with stillbirth (OR 2.54, 95% CI 1.33–4.83) and busy times that were ‘longer than before’ were associated with decreased odds of late stillbirth (OR 0.23, 95% CI 0.12–0.46). We found no association between late stillbirth and fetal movement patterns related to maternal hunger or consumption of food or drinks. (Supplementary Table 2).

**Figure 2:**
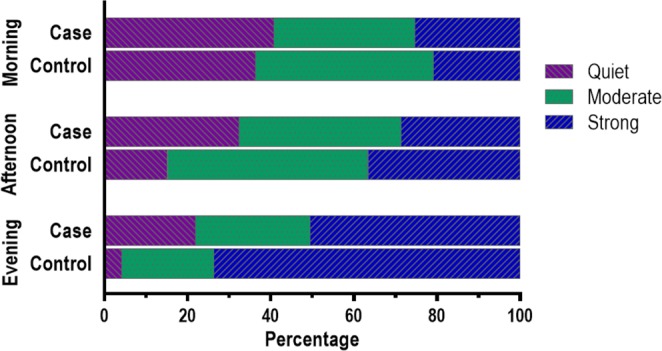
Fetal movement quality, time of day, and late stillbirth.

There were significant differences between cases and controls in terms of diurnal fetal movement pattern (Fig. 2). There was no difference between cases and controls in fetal movements in the morning. However, quiet or light movement in the afternoon (OR 2.63, 95% CI 1.5–4.58) and in the evening (OR 4.25, 95% CI 1.93–9.37) were associated with late stillbirth. Perception of strong movement in the evening (OR 0.55, 95% CI 0.33–0.93) or at night time (OR 0.44, 95% CI 0.26–0.74) was associated with decreased odds of late stillbirth. (Supplementary Table 3).

In multivariable analysis, with adjustment for potential confounders, busy times that were ‘longer than before’ were associated with reduced odds of late stillbirth (aOR 0.23 (0.11–0.47, p < 0.0001) and ‘quiet or light’ movement in the evening was associated with an almost 4-fold increased odds of late stillbirth (aOR 3.82, 95% CI 1.57–9.31, p < 0.0001) (Table 4).

**Table 4:** Fetal movement strength and pattern and odds of late stillbirth.

Fetal movement variable	Cases N = 101	Controls N = 333	Univariable Odds Ratio (95% Confidence Interval)	p	Multivariable Odds Ratio (95% Confidence Interval)	p
**In the last two weeks on average how long did these ‘busy times’ last?**						
Longer than before	11 (10.5)	125 (36.8)	0.23 (0.12–0.46)	<0.0001	0.23 (0.11–0.47)	<0.0001
About as long as before	72 (68.6)	191 (56.3)	reference		reference	
Shorter than before	22 (20.9)	23 (6.8)	2.54 (1.33–4.83)		1.89 (0.91–3.96)	
**During the evening**						
Quiet or subtle movement	23 (21.9)	14 (4.1)	4.25 (1.93–9.37)	<0.0001	3.82 (1.57–9.31)	<0.0001
Moderate movement	29 (27.6)	75 (22.2)	reference		reference	
Strong movements (including jumps or startles)	53 (50.5)	249 (73.7)	0.55 (0.33–0.93)		0.62 (0.35–1.11)	

Data are n (%). Adjusted for; parity, employment, body mass index, infant sex, and gestation. All fetal movement variables in table are included in model.

## Discussion

Understanding alterations in patterns of fetal movements prior to fetal death is clinically important as our study and others have shown that the most common presentation when the fetus has died is decreased fetal movements^14,15^. Our study has two novel and clinically relevant findings about fetal movement patterns prior to fetal demise. First, we found that there is a strong diurnal pattern in perceived fetal movement strength, and women who perceived their fetus to be quiet in the evening have an almost four-fold increased odds of late stillbirth. Second, we found that maternal perception of increasing length of fetal movement busy times over the past two weeks is associated with a four-fold reduction in odds of late stillbirth. In keeping with previous reports, we also found that decreased strength of fetal movements, decreased frequency of fetal movements and absence of hiccups are associated with stillbirth^13,14^.

Maternal perception of decreased fetal movements prior to fetal demise was first observed in the 1970s, following which fetal movement monitoring was proposed as a method of fetal surveillance to prevent stillbirth^16^. The most common method evaluated has been daily fetal movement counts^1^, but this has been shown to be ineffective for prevention of stillbirth in high-quality studies^17^. In recent years, emphasis has shifted to prioritizing the subjective impression of decreased or altered fetal movements on the part of the mother over any numeric definition^3^. However, simply promoting subjective awareness of fetal movements may not be useful. For example, pregnant women have reported being unsure what is normal and receiving vague or conflicting information about what they should be feeling^18^. Thus, the subjective impression method may lack specificity and has yet to prove useful for preventing stillbirths in high-quality studies^10^.

One method that has shown promise is Moore and Piacquadio’s investigation of ‘evening only’ fetal movement monitoring, given that the fetus is most likely to be active at this time^19^. In a prospective evaluation of a fetal movement screening programme designed to prevent stillbirth, women were instructed to count fetal movements between the hours of 7 and 11 pm and present that evening if they were unable to count 10 fetal movements in 2 hours. Moore and Piacquadio reported a significant (p < 0.01) reduction in stillbirths when using evening-only fetal movement screening. Our study provides further evidence that maternal monitoring of fetal movements specifically in the evening has potential utility in stillbirth prevention programmes. However, this would need to be assessed in large prospective studies that also consider possible unwanted effects such as excessive intervention.

Maternal perception of increased fetal movement in the evening has been reported previously^20–22^. The phenomenon is often ascribed to maternal inattention to fetal movements during the day or improved perception later in the day due to seated or reclined maternal positions^1,23^. However, perception of increased fetal movement in the evening has been demonstrated, even when accounting for maternal position^24^. In addition, ultrasound and chrono-biological studies have independently reported a diurnal fetal movement pattern characterized by increased fetal movement in the evening and greater likelihood of quiescence in the morning^25–27^. Our finding of a substantially increased likelihood of stillbirth with perception of quiet fetal movement in the evening further supports the presence of a true fetal diurnal movement pattern and absence of this pattern being indicative of fetal compromise.

There has been increasing interest in maximising effectiveness of healthcare treatments through consideration of circadian rhythms and optimized timing of interventions^28^. Screening and assessment of fetal movements is one area that might benefit from such an approach. Non-stress tests (NST) are more likely to be reactive when carried out at 9 pm compared to 9 am^29^, and biophysical profile scores are higher when conducted in the evening, due in part to a significant increase in fetal movement later in the day^30^. Thus fetal assessment may be more efficient in the evening due to the greater likelihood that the fetus will be in an active state at this time.

Pregnant women sometimes express that they are unsure what to monitor when it comes to fetal movement frequency, including how to regard clusters of movements^18^. We have shown that maternally perceived clusters of movements or busy times of the same or increasing length are associated with reduced odds of stillbirth. Busy times are likely to be indicative of active fetal behavioural state which is known to increase in length as pregnancy advances^31^. It has long been acknowledged that alterations in fetal movement quality precede a reduction in quantity of movements when fetal condition is deteriorating^32^. This may explain the sensitivity of maternal observation of fetal movement changes in predicting adverse outcome.

Ultrasound studies have shown that fetal movements are more readily detected by mothers if the movements are strong, of longer duration, involve more fetal body parts, or occur when the fetal heart pattern indicates an active fetal state^33–35^. Thus, quality of fetal movements influences perceived frequency. Incorporating perceived quality of fetal movements into screening programmes may prove more effective than observation of frequency alone.

Updating patient education to include information about the pattern of increased fetal movements in the evening may help pregnant women to better appreciate their fetus’ typical movement pattern and to seek timely assessment for fetal movement concerns, rather than delay until the following day. Antenatal information that reduces delays in DFM presentation has been shown to be effective in reducing stillbirths^9^. A focus on diurnal pattern would seem unlikely to result in excessive presentation given that just 4% of control women in our study perceived fetal movements to be quiet in the evening, compared to 14% who experienced decreased frequency.

The association between maternal perception of fetal hiccups and decreased odds of stillbirth has been reported previously^13,14^. Our data add further support to this association demonstrating that in women with daily perception of fetal hiccups there is a three-fold reduction in odds of stillbirth. Although it has been speculated that perception of fetal hiccups might indicate a cord accident^36^, hiccups are generally considered a normal aspect of fetal behaviour^37^. The hiccup phenomenon appears to universal in mammals although its’ origin and purpose remain a matter of speculation. Hiccups are more frequent in fetal and infant life than in adult life^38^. Fetal hiccups have been variously hypothesised to have a role in; developing respiratory muscles, preparing for suckling, and regulating amniotic fluid volume in early gestation^38,39^. Furthermore, central control of the hiccup reflex arc is in the brainstem^39^, suggesting presence of the hiccup reflex in the fetus may indicate normal neurological function. In a study of vibro-acoustic stimulation and hiccups during NST in 342 subjects, it was noted that fetal hiccups were absent in all non-reactive NST, also suggesting that absence of hiccups may be an indicator of fetal compromise^40^.

A strength of this study was the case-control design allowing for collection of detailed information about perceived fetal movements prior to fetal death from recently bereaved mothers and comparison with controls at similar gestation. The case-control design represents a practical approach investigating fetal movements in women with stillbirth, given that late stillbirth is a rare pregnancy outcome (3/1000 births) making a prospective cohort study impractical. Matching of stillbirth cases with controls by gestation and location minimises selection bias due to demographic factors. In addition, women with stillbirth were interviewed relatively soon after their stillbirth: within six weeks and at a median of 24 days, a time period in which bereaved parents are likely to accurately recall events surrounding the death of a baby^41^.

An acknowledged limitation of case-control study design is potential for recall bias. We took measures to minimise this risk by using a structured questionnaire to gather the fetal movement data. Midwife interviewers were trained to administer the questionnaire and instructed to ask the questions of both cases and controls exactly as written. To ensure women’s comfort with responding to questions, interviewers began the section of detailed fetal movement questions with the words ‘there are no right or wrong answers to any of these questions’. We anticipated that women may ask midwife interviewers for their opinion on factors being investigated in the study and instructed interviewers to respond that ‘evidence was inconclusive and that is why we are carrying out this study’. Use of midwife interviewers to administer the questionnaire also ensured sensitivity to vulnerable participants during interview. Feedback from participants about involvement with the study was overwhelmingly positive. With the exception of BB and RC, who conducted a small portion of the interviews, midwife interviewers had no role in generating the hypotheses being explored, or the subsequent data analysis, thus reducing the risk of interviewer inference.

Whilst risk of bias in case-control studies cannot be completely eliminated, it should be noted that some fetal movement variables explored in this study were novel and neither participants or interviewers could have anticipated any association with stillbirth. Notably, increased fetal movements in the evening and the association between fetal hiccups and stillbirth are not well known. Another possible limitation is the time delay between fetal death and interview in cases. This may have influenced accuracy of recall by cases; however, this would not be systematically biased toward the exposure^41,42^. Furthermore, the data in relation to fetal movement strength, frequency and hiccups closely resemble findings from a similar study conducted in the Midlands and North of England^13^, suggesting the findings are generalizable to other populations.

This study confirms the significance of maternal perception of decreases in strength and frequency of fetal movements and association with stillbirth. We have also shown that maternal perception of increasing strength of fetal movements, multiple episodes of movements that are more vigorous than usual, perception of fetal hiccups and busy times of the same or increasing length are all reassuring features. Of all fetal movement variables considered in this study, maternal perception of fetal quiescence in the evening had the strongest association with stillbirth. Investigation of approaches to fetal movement screening in future should take into consideration the diurnal pattern of increased fetal movement in the evening.

## Supplementary information

Supplementary Tables

## Data Availability

Due to ethical restrictions, data will be made available on request to Professor McCowan (l.mccowan@auckland.ac.nz) and subject to receiving appropriate New Zealand ethical approval. The Multi Centre Stillbirth Study research team is keen to promote collaboration with other researchers and to see our database used in studies that comply with New Zealand ethical regulations. Contact information for NZ Ethics Committee: Email: hdecs@moh.govt.nz; Postal address: Ministry of Health, Health and Disability Ethics Committees, PO Box 5013, Wellington 6140, New Zealand; Attention: Ethics Committees Manager for Protection, Regulation and Assurance.
