# Supplementary material for: Association between maternally perceived quality and pattern of fetal movements and late stillbirth
Source: Sci Rep. 2019 Jul 8;9:9815. doi: 10.1038/s41598-019-46323-4 (PMC6614481; doi:10.1038/s41598-019-46323-4)
Supplement: Supplementary file 1 — Supplementary Tables [file 41598_2019_46323_MOESM1_ESM.docx]

**Title**: Association between maternally perceived quality and pattern of fetal movements and late stillbirth

**Authors:** Bradford, Billie F. MMid^a^, Cronin Robin S. MMid^a^, McCowan, Lesley M. PhD^a^, McKinlay, Christopher J. PhD^b,c,d^, Mitchell, Edwin A. DSc^b^, Thompson, John M.D. PhD^a,b^

**Author Information**

^a^Department of Obstetrics and Gynaecology, Faculty of Medical and Health Sciences, University of Auckland, Auckland, New Zealand.

^b^Department of Paediatrics: Child and Youth Health, Faculty of Medical and Health Sciences, University of Auckland, Auckland, New Zealand.

^c^Liggins Institute, University of Auckland, Auckland, New Zealand.

^d^Kidz First Neonatal Care, Counties Manukau Health, Auckland, New Zealand

**Supplementary information**

**Supplementary Table 1: Fetal movement strength and frequency combinations and late stillbirth**

| **Strength** | **Frequency** | **Stillbirths N=164** | **Controls N=568** | **Univariable OR (95%CI)** |
| --- | --- | --- | --- | --- |
| Increase | Increase | 12 (7.3%) | 192 (33.8%) | 0.12 (0.14-0.49) |
| Increase | Decrease | 6 (3.6%) | 25 (4.4%) | 0.48 (0.19-1.24) |
| Increase | Same | 14 (8.5%) | 108 (19.0%) | 0.26 (1.14-0.49) |
| Increase | Unsure | 0 | 5 (0.9%) | - |
|  |  |  |  |  |
| Decrease | Increase | 3 (1.8%) | 0 | undefined |
| Same | Increase | 6 (3.6%) | 29 (5.1%) | 0.42 (0.16-1.06) |
| Unsure | Increase | - | - | undefined |
|  |  |  |  |  |
| Decrease | Decrease | 40 (24.4%) | 28 (4.9%) | 2.88 (1.62-5.11) |
| Same | Decrease | 12 (7.3%) | 27 (5.1%) | 0.89 (0.42-1.89) |
| Unsure | Decrease | 4 (2.4%) | 3 (0.5%) | 2.69 (0.58-12.40) |
|  |  |  |  |  |
| Decrease | Unsure | 0 | 1 (0.2%) | undefined |
| Same | Unsure | 2 (1.2%) | 3 (0.5% | 1.34 (0.22-8.26) |
| Unknown | Same | 1 (0.6%) | 9 (1.6%) | 0.22 (0.03-1.81) |
| Unsure | Unsure | 0 | 1 (0.9%) | - |
|  |  |  |  |  |
| Decrease | Same | 4 (2.4%) | 16 (2.8%) | 0.50 (0.16-1.57) |
| Same | Same | 60 (36.6%) | 121 (21.3%) | reference |

One control subject had missing data.

**Supplementary Table 2: Fetal movement strength and pattern and late stillbirth**

| During the last two weeks,  please comment on your baby’s movements… | Cases N=112 | Controls  N=345 | p |
| --- | --- | --- | --- |
| **How many ‘busy times’ did your baby have in a day?** | | | |
| 0-2 | 37 (33.6) | 82 (23.8) | 0.13 |
| 3-9 | 63 (57.3) | 225 (65.4) |  |
| 10+ | 10 (9.1) | 37 (10.8) |  |
| **On average, how long did these ‘busy times last?** | | | |
| Longer than before | 11 (10.5) | 125 (36.8) | <0.0001 |
| About as long as before | 72 (68.6) | 191 (56.3) |  |
| Shorter than before | 22 (20.9) | 23 (6.8) |  |
| **When you first wake (before rising out of bed)** | | | |
| Quiet or light | 42 (42.4) | 148 (44.4) | 0.29 |
| Moderate | 29 (29.3) | 115 (34.5) |  |
| Strong or jumps | 28 (28.3) | 70 (21.0) |  |
| **During the morning** | | | |
| Quiet or light | 42 (40.8) | 122 (36.3) | 0.27 |
| Moderate | 35 (34.0) | 144 (42.8) |  |
| Strong or jumps | 26 (25.2) | 70 (20.8) |  |
| **During the afternoon** | | | |
| Quiet or light | 34 (32.4) | 51 (15.2) | 0.0005 |
| Moderate | 41 (39.0) | 161 (48.2) |  |
| Strong or jumps | 30 (28.6) | 123 (36.6) |  |
| **During the evening** | | | |
| Quiet or light | 23 (21.9) | 14 (4.1) | <0.0001 |
| Moderate | 29 (27.6) | 75 (22.2) |  |
| Strong or jumps | 53 (50.5) | 249 (73.7) |  |
| **Night time (including bedtime)** | | | |
| Quiet or light | 19 (18.4) | 26 (7.7) | <0.0001 |
| Moderate | 30 (29.1) | 61 (18.1) |  |
| Strong or jumps | 54 (52.4) | 250 (74.2) |  |
| **Before a usual meal time** | | | |
| Quiet or light | 55 (63.2) | 170 (59.8) | 0.65 |
| Moderate | 22 (25.3) | 92 (24.6) |  |
| Strong or jumps | 10 (11.5) | 44 (15.5) |  |
| **When you are hungry** | | | |
| Quiet or light | 48 (54.5) | 149 (53.2) | 0.81 |
| Moderate | 21 (23.9) | 76 (27.1) |  |
| Strong or jumps | 19 (21.6) | 55 (19.6) |  |
| **While you are eating** | | | |
| Quiet or light | 50 (56.2) | 176 (57.7) | 0.66 |
| Moderate | 26 (29.2) | 76 (24.9) |  |
| Strong or jumps | 13 (14.6) | 53 (17.9) |  |
| **Within 15 minutes of eating** | | | |
| Quiet or light | 34 (39.1) | 114 (37.9) | 0.58 |
| Moderate | 32 (36.8) | 98 (32.6) |  |
| Strong or jumps | 21 (24.1) | 89 (29.6) |  |
| **An hour after eating** | | | |
| Quiet or light | 43 (51.8) | 114 (40.0) | 0.06 |
| Moderate | 22 (26.5) | 115 (40.3) |  |
| Strong or jumps | 18 (21.7) | 56 (19.6) |  |
| **When you are walking around at home or work** | | | |
| Quiet or light | 51 (53.1) | 193 (60.1) | 0.47 |
| Moderate | 31 (32.3) | 89 (27.7) |  |
| Strong or jumps | 14 (14.53) | 39 (12.1) |  |
| **When you are standing in one spot** | | | |
| Quiet or light | 49 (51.0) | 181 (55.9) | 0.48 |
| Moderate | 29 (30.2) | 98 (30.25) |  |
| Strong or jumps | 18 (18.7) | 45 (13.9) |  |
| **When you are sitting quietly** | | | |
| Quiet or light | 24 (23.5) | 60 (17.9) | 0.42 |
| Moderate | 33 (32.3) | 123 (36.7) |  |
| Strong or jumps | 45 (44.1) | 152 (45.4) |  |
| **When you lie on your side** | | | |
| Quiet or light | 34 (34.7) | 80 (24.2) | 0.05 |
| Moderate | 36 (36.7) | 117 (35.4) |  |
| Strong or jumps | 28 (28.6) | 133 (40.3) |  |
| **When you have a cold drink (water, juice, fizzy or other cold liquid)** | | | |
| Quiet or light | 34 (40.9) | 104 (35.2) | 0.57 |
| Moderate | 21 (25.3) | 89 (30.2) |  |
| Strong or jumps | 28(33.7) | 102 (34.6) |  |
| **When you sit in a cramped position (eg in car, or sitting with knees pulled up)** | | | |
| Quiet or light | 30 (39.5) | 103 (35.0) | 0.74 |
| Moderate | 24 (31.6) | 95 (32.3) |  |
| Strong or jumps | 22 (31.6) | 96 (32.6) |  |
| **When you rub or prod parts of baby** | | | |
| Quiet or light | 31 (30.4) | 89 (27.1) | 0.74 |
| Moderate | 30 (29.4) | 108 (32.9) |  |
| Strong or jumps | 41 (40.2) | 131 (39.9) |  |
| **When there is an unexpected loud noise (e.g. dogs barking, shouting, music or banging)** | | | |
| Quiet or light | 40 (53.3) | 113 (45.7) | 0.05 |
| Moderate | 22 (29.3) | 56 (22.7) |  |
| Strong or jumps | 13 (17.3) | 78 (31.6) |  |

Data are n (%)

**Supplementary Table 3: Fetal movement quality and odds ratio for late stillbirth**

|  | Cases  N=112 | Controls  N=345 | Odds Ratio (95% Confidence Interval) |
| --- | --- | --- | --- |
| **In the last two weeks on average how long did these ‘busy times’ last?** | | | |
| Longer than before | 11 (10.5) | 125 (36.8) | 0.23 (0.12-0.46) |
| About as long as before | 72 (68.6) | 191 (56.3) | reference |
| Shorter than before | 22 (20.9) | 23 (6.8) | 2.54 (1.33-4.83) |
| **During the afternoon** | | | |
| Quiet or light | 34 (32.4) | 51 (15.2) | 2.63 (1.5-4.58) |
| Moderate | 41 (39.0) | 162 (48.2) | reference |
| Strong or jumps | 30 (28.6) | 123 (36.6) | 0.96 (0.57-1.63) |
| **During the evening** | | | |
| Quiet or light | 23 (21.9) | 14 (4.1) | 4.25 (1.93-9.37) |
| Moderate | 29 (27.6) | 75 (22.2) | reference |
| Strong or jumps | 53 (50.5) | 249 (73.7) | 0.55 (0.33-0.93) |
| **Night time including bedtime** | | | |
| Quiet or light | 19 (18.4) | 26 (7.7) | 1.49 (0.71-3.1) |
| Moderate | 30 (29.1) | 61 (18.1) | reference |
| Strong or jumps | 54 (52.4) | 250 (74.2) | 0.44 (0.26-0.74) |
| **When you lie on your side** | | | |
| Quiet or light | 34 (34.7) | 80 (24.2) | 1.38 (0.79-2.39) |
| Moderate | 36 (36.7) | 117 (35.4) | reference |
| Strong or jumps | 28 (28.6) | 133 (40.3) | 0.68 (0.39-1.19) |
| **An hour after eating*** | | | |
| Quiet or light | 43 (51.8) | 114 (40.0) | 1.98 (1.1-3.5) |
| Moderate | 22 (26.5) | 115 (40.3) | reference |
| Strong or jumps | 18 (21.7) | 56 (19.6) | 1.68 (0.83-3.38) |
| **Unexpected loud noise*** | | | |
| Quiet or light | 40 (53.3) | 113 (45.7) | 0.9 (0.49-1.66) |
| Moderate | 22 (29.3) | 56 (22.7) | reference |
| Strong or jumps | 13 (17.3) | 78 (31.6) | 0.42 (0.19-0.91) |

* Variables with >10% missing data. Data are n (%).
